# What-if effects: A tale of entangled covariates

**DOI:** 10.1016/j.patter.2026.101616

**Published:** 2026-09-11

**Authors:** Zhaowei Yu, Yong Zhang

**Affiliations:** 1 State Key Laboratory of Cardiovascular Diseases and Medical Innovation Center, Institute for Regenerative Medicine, Department of Neurosurgery, Shanghai East Hospital, Shanghai Key Laboratory of Signaling and Disease Research, Frontier Science Center for Stem Cell Research, School of Life Sciences and Technology, Tongji University, Shanghai, 200092, China

## Abstract

Single-cell epigenomic studies promise to reveal how disease states, donor traits, environmental exposures, and technical factors shape cellular regulatory programs, yet these variables are often highly collinear and difficult to disentangle, particularly in human cohorts. The recent study by Møller and Madsen in *Patterns* introduces DeepDive, a probabilistic deep-learning framework that disentangles known covariate effects and residual variations in single-nucleus ATAC-seq data, enabling counterfactual “what-if” analyses of chromatin accessibility.

## Main text

Single-cell technologies have transformed cohort-scale biology by enabling researchers to examine how disease status, donor traits, environmental exposures, developmental programs, and technical factors shape molecular cell states at single-cell resolution.^1^^,^^2^ Yet in observational human cohorts, these variables rarely vary independently. Disease labels may be entangled with age, sex, obesity, genetic background, medication history, sample quality, or batch, making it difficult to assign observed molecular differences to a specific biological factor.^1^^,^^3^ This problem is especially challenging in single-cell epigenomics. Single-nucleus ATAC sequencing (snATAC-seq) profiles are sparse and high dimensional, and chromatin accessibility reflects both cell identity and regulatory responses to donor-level, environmental, and technical variables. When these variables are correlated, apparent disease-associated peaks may partly reflect accompanying biological or technical factors rather than disease itself.^4^ Existing snATAC-seq workflows are powerful for preprocessing, peak calling, clustering, integration, and differential accessibility analysis,^5^^,^^6^ and recent representation-learning methods provide useful foundations for disentangling single-cell variation.^7^^,^^8^ However, these approaches, together with conventional differential analysis frameworks,^9^ are not primarily designed for robust covariate-specific effect estimation under such conditions. Thus, a central challenge for cohort-scale single-cell epigenomics is to move from asking “what differs between groups?” to asking “what would the same cellular regulatory state look like if one covariate were changed while the others were held fixed?”

In a recent *Patterns* paper, Møller and Madsen address this challenge with DeepDive, a semi-supervised probabilistic framework for disentangling covariate effects in single-cell epigenomic data. DeepDive represents annotated covariates through covariate-specific embeddings and uses a residual variational component to capture remaining sources of variation. Through adversarial training, the model discourages known covariate information from being encoded in the residual latent space, thereby reducing leakage between annotated and unannotated sources of variation. A multi-decoder design further provides uncertainty estimates from reconstructed accessibility profiles, supporting feature-level effect estimation and differential accessibility analysis. This architecture enables counterfactual “what-if” analysis: after training, one can hold the cellular background fixed while virtually changing a covariate of interest. The resulting difference in reconstructed chromatin accessibility provides a model-based estimate of how that covariate is associated with regulatory changes. In this way, DeepDive moves beyond correcting unwanted variation toward model-based interpretation of covariate-associated regulatory effects.

DeepDive’s strength is illustrated across three common difficulties in cohort-scale single-cell epigenomic analysis. It can recover meaningful structure when key annotations are missing, reduce signal leakage when covariates are correlated, and reconstruct chromatin accessibility for covariate combinations not observed during training. In simulations with moderate collinearity, DeepDive recovered substantially more true differential features than Wilcoxon rank-sum testing, illustrating how conventional group-wise comparisons can lose power when covariate effects leak across correlated variables. DeepDive also supports practical “what-if” use cases, including batch integration and prediction of missing donor covariates. In these settings, DeepDive performs competitively with specialized integration or statistical methods while additionally providing covariate disentanglement and counterfactual inference, making it useful not only for correcting confounding variation but also for interpreting how individual biological or technical factors shape single-cell-resolved epigenomes.

The biological utility of DeepDive is illustrated in human pancreatic islet snATAC-seq data, where BMI-related obesity effects and type 2 diabetes are clinically entangled.^1^^,^^3^ By jointly modeling biological and technical covariates, DeepDive first prioritized beta cells as the cell type most strongly affected by both BMI and diabetes status and then separated the accessibility features associated with each covariate. This interpretation was supported by genetic enrichment analysis, in which BMI-associated beta-cell peaks were enriched for obesity-linked variants but not type 2 diabetes variants. Beyond identifying affected cell types and regulatory regions, DeepDive also enabled counterfactual analysis of beta-cell substates, suggesting that diabetes status had the strongest predicted effect on the beta-1/beta-2 balance, with smaller contributions from HbA1c, BMI, and age. Finally, by comparing motif activity in reconstructed and counterfactual accessibility profiles, DeepDive nominated candidate regulators of the beta-cell state transition, with counterfactually supported regulators showing stronger enrichment for diabetes-related genetic evidence and glucose-stimulated insulin secretion associations. Together, this application shows that DeepDive can move from covariate disentanglement to cell-type prioritization, regulatory interpretation, and disease-relevant mechanism discovery in complex human cohorts.

As with any counterfactual model, DeepDive’s predictions should be interpreted as model-based hypotheses rather than direct experimental interventions. The framework assumes that annotated covariates are informative, at least partially separable, and combinable in an additive latent space. Therefore, hierarchical, redundant, or completely collinear annotations may remain difficult to resolve. Counterfactual predictions are also most reliable when they remain close to the support of the observed data, whereas extrapolation to distant covariate combinations requires caution. Future extensions could relax some of these assumptions, for example through interaction-aware or multiplicative fusion, and could connect counterfactual chromatin changes to gene-expression consequences through joint modeling of paired single-cell RNA-seq and ATAC-seq data. Incorporating genotype, regulatory sequence, perturbation screens, or prior gene-regulatory networks may further help distinguish statistical disentanglement from causal mechanism.

Despite these challenges, DeepDive represents an important step toward interpretable modeling of complex human single-cell cohorts. Its key conceptual contribution is to treat covariates not simply as nuisances to regress out or labels for stratified comparison but as structured explanatory variables whose effects can be estimated, compared, and virtually perturbed. This shift is particularly useful for disease atlases, developmental studies, and perturbation datasets, where biological variables are rarely independent but are precisely the variables researchers seek to understand. By helping single-cell epigenomics ask not only what is observed but also what might have been observed under a different biological condition, DeepDive provides a practical framework for turning entangled cohort variation into testable regulatory hypotheses.

## Acknowledgments

This work is supported by the National Natural Science Foundation of China (32325012, 32488101) and the Science and Technology Commission of Shanghai Municipality (23JS1401200, 26XF3200400).

## Declaration of interests

The authors declare no competing interests.
